# Patterns of Endodontic Practice and Technological Uptake Across Training Levels in Spain and Latin America: Results from a Multicountry Survey of 1358 Clinicians

**DOI:** 10.3390/dj13120558

**Published:** 2025-11-27

**Authors:** Rocío Piñas-Alonzo, Alejandro R. Pérez, José Aranguren, Gaya C. S. Vieira, Juan Carlos Paz, Juan Saavedra, Jenny Guerrero Ferreccio, Simone Grandini, Giulia Malvicini

**Affiliations:** 1 Department of Endodontics, Rey Juan Carlos University, 28922 Madrid, Spain; 2 Surpreendente Research Group, 4400-239 Vila Nova de Gaia, Portugal; 3 Private Practice, Mexico City 11520, Mexico; 4 Private Practice, Caracas 1080, Venezuela; 5 Graduate Endodontics Program, School of Dentistry, Universidad Catolica de Santiago de Guayaquil (UCSG), Guayaquil 090615, Ecuador; jenny.guerrero01@cu.ucsg.edu.ec; 6 School of Dentistry, Universidad Catolica de Santiago de Guayaquil (UCSG), Guayaquil 090615, Ecuador; 7 Unit of Endodontics and Restorative Dentistry, Department of Medical Biotechnologies, University of Siena, 53100 Siena, Italy

**Keywords:** endodontic practice, Latin America, survey, Spain, technology adoption

## Abstract

**Background/Objectives**: The objective of this study was to investigate current endodontic practice patterns and the adoption of newer technologies among dentists, endodontic specialists, and postgraduate students in Spain and Latin America. **Methods:** A cross-sectional survey was conducted using a structured 30-item questionnaire covering demographics, training, technology adoption (NiTi instrumentation, magnification, CBCT, irrigation adjuncts, bioceramic sealers), obturation techniques, irrigant selection, and clinical procedures. The survey was distributed through a professional Instagram account and WhatsApp groups of dentists, specialists, and postgraduate students. Participation was voluntary, anonymous, and restricted to qualified professionals. Data were collected via Google Forms, cleaned, and grouped into Spain, Mexico, Venezuela, Colombia, Southern Cone & Andes (Argentina, Chile, Peru, Ecuador, Bolivia), and other countries. Descriptive statistics were calculated, and intergroup comparisons were performed using Chi-square or Fisher’s exact tests with Bonferroni correction (*p* < 0.05). Multiple regression analyses were performed. **Results:** A total of 1358 valid responses were analyzed, distributed as follows: Spain (219), Mexico (353), Venezuela (162), Colombia (108), Southern Cone & Andes (260), and other countries (256). Most respondents (62.8%) had ≤10 years of experience, and 61.2% reported postgraduate training. Loupes (55.4%) were the most frequent magnification system, followed by microscopes (18.6%). Sodium hypochlorite (98.3%) was the irrigant of choice, commonly used with EDTA (83.5%) and, to a lesser extent, chlorhexidine (33.4%). Sonic (83.2%) and ultrasonic (52.9%) activation were frequent. Bioceramic sealers were used by 18.9%, while calcium hydroxide medication was applied by 37.4%. Specialists and master-level clinicians showed greater use of rotary NiTi systems, CBCT, magnification, and bioceramic sealers, whereas general practitioners relied more on manual instrumentation and single-cone obturation. Success was mainly verified by combined clinical and radiographic evaluation (86.7%). Spain demonstrated higher adoption of microscopes, warm vertical compaction, and CBCT. **Conclusions:** Core practices such as sodium hypochlorite irrigation and rubber dam use were widespread, while advanced technologies and irrigant protocols varied with training level and region. Continuous education remains essential to promote evidence-based practice and reduce disparities in endodontic innovation.

## 1. Introduction

Endodontics has undergone significant technological and conceptual advances over the past three decades [1,2]. The widespread introduction of nickel–titanium (NiTi) rotary and reciprocating instruments, enhanced irrigation strategies including ultrasonic and sonic activation, the availability of cone-beam computed tomography (CBCT), and the development of bioceramic sealers and repair materials have reshaped clinical protocols in endodontics [1,3,4,5,6]. At the same time, the use of magnification through loupes or microscopes has become increasingly recommended to improve precision during root canal procedures [7].

Despite the evidence supporting these innovations, the degree to which they have been adopted in routine practice varies across regions and among practitioners with different training backgrounds [1,8,9]. Surveys of endodontic practice have provided valuable insights into these patterns, highlighting discrepancies between clinical guidelines and everyday practice [1,10]. For example, studies in the United States and Europe have demonstrated gradual but uneven adoption of newer technologies [11], while a recent global survey revealed considerable heterogeneity between continents [1]. However, Latin America remains largely underrepresented in this type of research, and data from Spain are scarce [1].

Given the cultural, economic, and educational links between Spain and Latin America, understanding current trends in these regions provides an opportunity to contextualize the diffusion of innovations within Latin America, complementing existing global surveys and highlighting where adoption differs substantially (e.g., CBCT usage and magnification) [1,12,13].

Identifying similarities and differences in the adoption of technologies such as NiTi instrumentation, magnification, CBCT, irrigation protocols, obturation techniques, and bioceramic sealers can help guide continuing education initiatives and research priorities.

Therefore, this study aimed to conduct a multicenter survey of endodontic practice in Spain and Latin America, analyzing trends in clinical decision-making and adoption of newer technologies across countries with the highest number of respondents.

## 2. Materials and Methods

### 2.1. Study Design

This study was designed as a cross-sectional survey of endodontic practice. Participation was voluntary, anonymous, and preceded by electronic informed consent [14].

### 2.2. Questionnaire Development

The questionnaire was constructed based on previous surveys of endodontic practice [1], with modifications to reflect contemporary technologies and clinical decision-making. It consisted of approximately 30 items covering four main domains: (i) demographic and training background, (ii) adoption of technologies (NiTi instrumentation, magnification, CBCT, irrigation adjuncts, bioceramic sealers), (iii) obturation techniques and irrigant selection, and (iv) additional clinical practice patterns (rubber dam usage, number of visits, use of calcium hydroxide, apical patency, follow-up).

### 2.3. Survey Distribution

The survey was disseminated between March 2023 and June 2023 through social media channels, including a professional Instagram account and WhatsApp groups of dentists, endodontic specialists, and postgraduate endodontic students. Participants were invited to complete the questionnaire and encouraged to share the survey link with colleagues. Missing data were handled by case-wise deletion for the affected variables, with complete response numbers reported in each table. Given the open nature of survey dissemination, no a priori sample size calculation was feasible; however, the large sample size (>1300 valid responses) provides sufficient statistical power for the primary descriptive aims.

### 2.4. Sample Validation

The questionnaire was developed by the authors and reviewed internally by three experienced endodontists for clarity and content validity. Prior to dissemination, the questionnaire was pre-tested with a small group of postgraduate students (*n* ≈ 10) to ensure clarity, logical flow, and approximate response time, and minor adjustments to wording were made based on feedback.

Inclusion criteria were dentists or postgraduate students engaged in clinical endodontic practice. Incomplete or inconsistent responses were excluded from the analysis. To further ensure the validity of the sample, the questionnaire included a mandatory screening item regarding professional status (general dentist, endodontic specialist, or postgraduate student in endodontics).

Only respondents who identified within these categories were retained. In addition, the survey link was disseminated exclusively through professional channels, including an Instagram account dedicated to endodontics and WhatsApp groups of dentists and postgraduate programs, which limited access to the target audience and minimized the risk of non-professional responses.

### 2.5. Data Management and Statistical Analysis

Data were collected through Google Forms and exported to Microsoft Excel (version 16.0, Microsoft Corporation, Redmond, WA, USA) for cleaning and processing. Country names were standardized, and respondents were grouped into the following categories: Spain, Mexico, Venezuela, Colombia, Southern Cone & Andes (Argentina, Chile, Peru, Ecuador, Bolivia), and other countries.

Descriptive statistics were reported as frequencies and percentages. Group comparisons were performed using Chi-square or Fisher’s exact test when expected frequencies were low, with Bonferroni correction applied for multiple testing and a significance level set at *p* < 0.05. Additionally, exploratory multivariate logistic regression analyses were performed for selected outcomes (bioceramic sealer use and CBCT adoption), adjusting for years of experience, country group, and training status. All analyses were conducted using SPSS version 26.0 (IBM Corp., Armonk, NY, USA) and Python version 3.11 (statsmodels library).

## 3. Results

### 3.1. Demographic Characteristics

**Figure 1:**
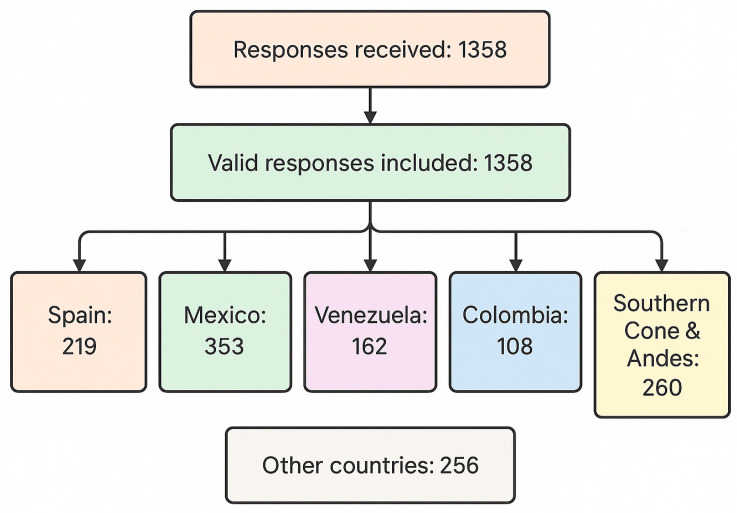
Demographic characteristics of the population under study.

A total of 1358 valid responses were analyzed, comprising 219 from Spain, 353 from Mexico, 162 from Venezuela, 108 from Colombia, and 260 from Argentina, Chile, Peru, Ecuador, and Bolivia (collectively referred to as the Southern Cone & Andes). Additionally, 256 responses were from other Latin American countries (Figure 1).

Demographic characteristics are presented in Table 1.

Most respondents reported less than 10 years of clinical experience, with 39.9% having 1–5 years and 22.9% having 6–10 years of practice. Specialists or dentists with structured postgraduate training represented the majority, as 32.3% had completed a ≥2-year master’s program and 28.9% a modular postgraduate course. Only 11.4% reported having undergraduate training only. Technology adoption and clinical practices are summarized in Table 2 and Table 3.

### 3.2. Magnification and Imaging

**Figure 2:**
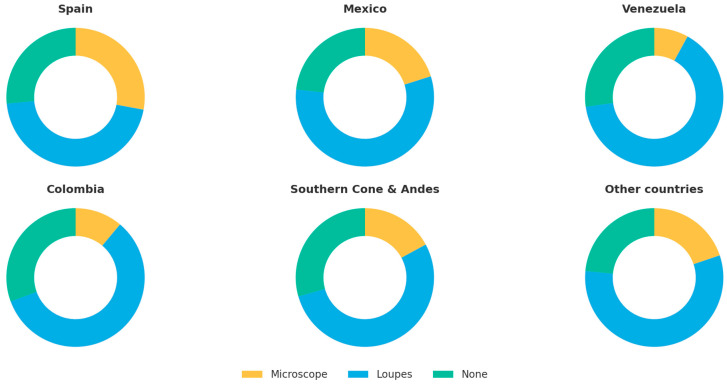
Magnification systems employed in different countries.

Loupes were the most frequently used system (55.4%), followed by the dental operating microscope (18.6%), while 26.1% did not use any magnification (Table 2 and Figure 2. The use of magnification strongly increased with training level: 87% of those with ≥2-year master’s programs employed some form of magnification (34% microscopes) compared with 71% among modular postgraduates, 45% of those trained through short courses, and 32% of general practitioners (Table 3).

### 3.3. Irrigants and Activation

**Figure 3:**
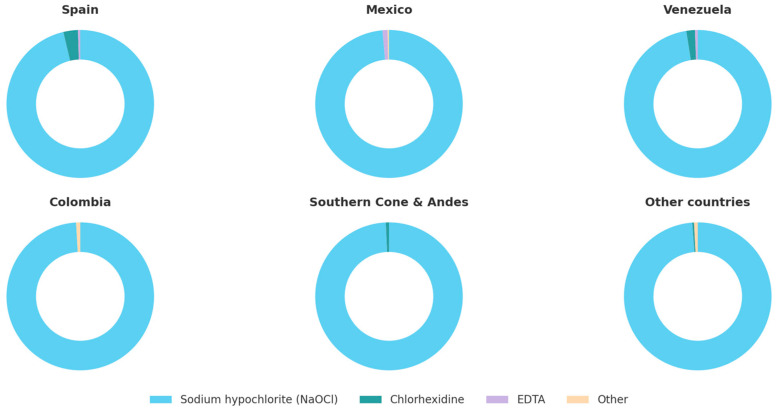
Irrigant choice by country group.

Sodium hypochlorite (NaOCl) was overwhelmingly the irrigant of choice (98.3%), usually at concentrations between 2.5 and 5.25%, confirming its role as the primary irrigant across all training categories (Table 2 and Table 3 and Figure 3). Lower concentrations (<0.5%) were rarely employed (3.8%), and 2.3% of respondents were unsure of the concentration used. Clinicians with advanced postgraduate education were more likely to employ higher NaOCl concentrations (≥2.5%).

EDTA was employed for smear layer removal by 84% of respondents, and chlorhexidine by 33%. Postgraduate-trained clinicians were significantly more likely to use EDTA and chlorhexidine as adjuncts, whereas non-postgraduates relied mainly on NaOCl alone.

Among those using chlorhexidine, the most frequent concentration was 0.12% (typically used as an adjunctive irrigant), reported by 10.8% of postgraduate students and 15.0% of participants with short-course training. The use of 0.05% solutions was limited (1.5–5.2%), while over 60% of respondents in all groups reported not using chlorhexidine at all.

Sonic activation was reported by 83.2% and ultrasonic activation by 52.9%; both methods were markedly more frequent among specialists (96% and 78%, respectively) than among undergraduate-level clinicians (<40%). Some country-level differences were observed, especially regarding the adoption of ultrasonic activation, which was notably higher in Mexico.

Negative-pressure irrigation was uncommon (4.0%), and 3.9% indicated not using any adjunctive system.

### 3.4. Instrumentation and Obturation

Combined use of manual and rotary instruments was predominant across all training levels (overall 89.9%), reflecting the widespread adoption of hybrid techniques. This trend was most pronounced among master’s graduates (90.9%) and modular postgraduate clinicians (86.3%), whereas a higher proportion of manual-only users was observed among general practitioners (37.4%) and those trained through short courses (27.9%). Rotary-only approaches were uncommon, with slightly higher adoption among postgraduate-trained respondents, indicating selective use in specific clinical scenarios rather than as a routine method.

**Figure 4:**
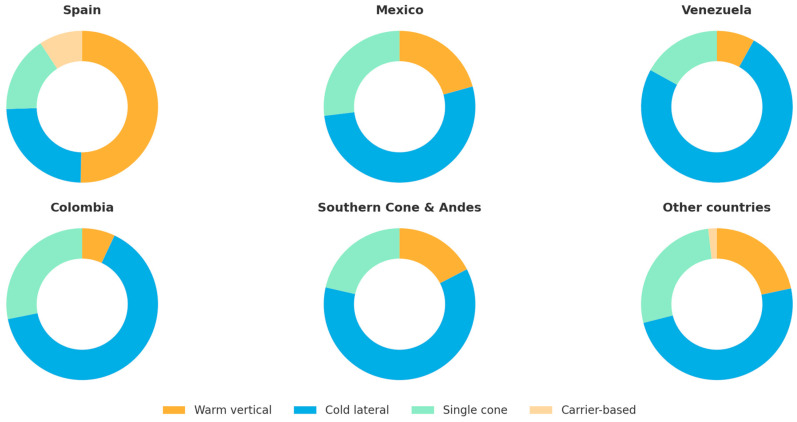
Obturation technique by country group.

Cold lateral condensation remained the most frequent obturation technique overall (34.0%), followed by warm vertical compaction (15.8%) and single-cone (15.5%). However, warm vertical compaction was preferred by >40% of specialists, compared with <10% of non-postgraduates. Moreover, some regional differences were observed as cold lateral condensation remained particularly prevalent in Venezuela and Colombia, while warm vertical compaction was more commonly employed in Spain (Figure 4).

**Figure 5:**
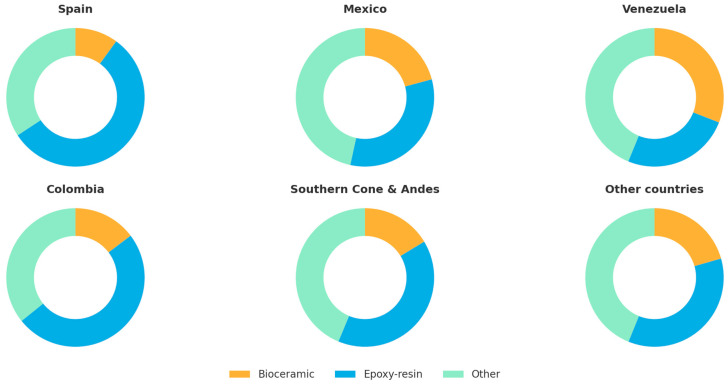
Types of sealers preferred in different countries.

In terms of sealer type (Table 2 and Table 3), epoxy-resin sealers were the most common overall, accounting for 82.0% of responses. Bioceramic sealers, on the other hand, accounted for 18.0% of responses. Their use was highest among master-level clinicians (≈19%) and modular postgraduates (≈17%), decreasing sharply among general practitioners (≈7%). The adoption of bioceramic sealers was more common in Venezuela than in other countries (Figure 5).

Exploratory logistic regression analyses (Table 4) confirmed that Venezuelan respondents were significantly more likely to report bioceramic sealer (OR 2.71, 95% CI: 1.43–5.12, *p* = 0.002).

### 3.5. CBCT Utilization

**Figure 6:**
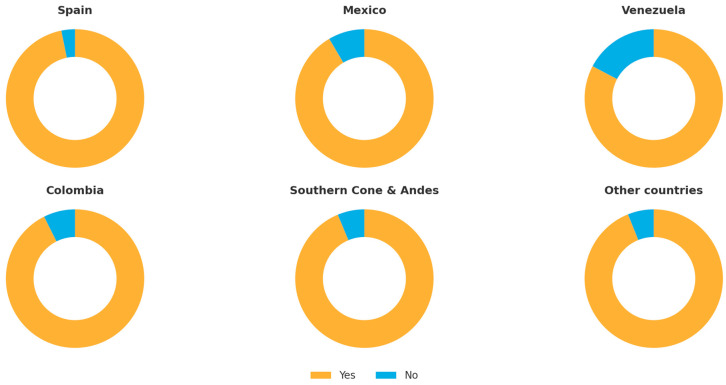
CBCT usage across different countries.

Overall, 91.3% of respondents reported using CBCT for specific indications such as complex anatomy, retreatments, and apical surgery (Table 2 and Table 3, and Figure 6). However, its integration varied substantially according to training level. Among respondents with a ≥2-year master’s degree, nearly all (96%) reported regular CBCT use, compared with 89% of modular postgraduate participants, 73% of those attending short courses or congresses, and only 62% of general practitioners or recent graduates. The frequency of CBCT use was also higher among specialists performing endodontics exclusively (over 90% within this subgroup), while occasional or selective use predominated among general clinicians.

When asked about the specific scenarios for CBCT indication, most clinicians (72.5%) reported using it for multiple or general purposes, including case planning, complex anatomy, retreatments, and surgical assessment. This pattern was most pronounced among master’s (78.8%) and current postgraduate students (77.9%), whereas a significantly lower proportion was observed among those with short-course training (47.9%) or only undergraduate education (52.3%). Conversely, lack of CBCT access or non-use was reported by up to 26.3% of participants in the courses/congress group and 15.5% of bachelor-level clinicians.

Multivariate analysis (Table 5) confirmed postgraduate education as the strongest predictor of CBCT utilization (OR = 3.7; 95% CI: 2.3–5.9; *p* < 0.001), even after controlling for years of experience and country of practice.

### 3.6. Rubber Dam Isolation

Secondary clinical practice patterns are presented in Table 6 and Table 7. Rubber dam use was reported by 92.7% of all respondents, confirming it as a standard component of endodontic practice across Spain and Latin America. Nevertheless, adherence varied markedly with educational background. Nearly all clinicians with a ≥2-year master’s program (98%) and 94% of modular postgraduates reported systematic rubber dam use, compared with 82% of those trained through short courses or congresses, and only 68% of respondents with undergraduate training only. The rate of consistent rubber dam application was also higher among those performing endodontics exclusively and among specialists working in academic or referral settings.

### 3.7. Inter-Appointment Procedures and Medicaments

Calcium hydroxide was used as an inter-appointment medicament by approximately 70% of respondents, although only 37% reported using it routinely in multi-visit treatments. Most clinicians applied calcium hydroxide selectively—primarily in cases of necrotic pulps, retreatments, or when suppuration was present—with this pattern reported by 45–53% of respondents across all training levels. Routine use of calcium hydroxide did not differ substantially among groups, ranging from 37.4% among ≥2-year master-trained clinicians to 46.3% among those whose training consisted mainly of short courses.

Single-visit root canal treatment was performed by 57.6% of respondents overall, but its adoption increased sharply with structured postgraduate education. A single-visit approach was reported by 75.4% of clinicians with a ≥2-year master’s degree, 64.1% of those currently enrolled in postgraduate programs, and 62.6% of those with modular postgraduate training, compared with 43.9% of clinicians with only a bachelor’s degree and 27.1% of those trained exclusively through short courses. Conversely, multiple-visit protocols were most prevalent among respondents without formal postgraduate training.

### 3.8. Apical Patency

Apical patency was maintained by 81.2% of respondents as part of their working length strategy. This approach was most consistently reported by postgraduate-trained clinicians (over 90%), especially those with ≥2-year master’s programs, whereas only 63% of general practitioners or dentists without advanced endodontic education routinely preserved patency. Clinicians with higher training levels were also more likely to combine electronic apex locators with radiographic verification and to use continuous irrigation during working length maintenance.

### 3.9. Follow-Up

Regarding follow-up, approximately half of all participants scheduled both clinical and radiographic reviews within the first year after treatment, whereas others limited review to cases with persistent symptoms. Postgraduate clinicians were significantly more likely to perform systematic radiographic follow-up and to use standardized recall intervals (Table 3).

### 3.10. Postoperative Pain and Fracture After RCT

Postoperative pain was generally considered uncommon across all groups. Overall, 86.4% of respondents reported it as uncommon, while 13.6% described it as common, and none indicated that it never occurs. The prevalence of pain did not differ significantly among training levels, although master-level clinicians more often attributed postoperative symptoms to preoperative inflammation or procedural complexity rather than technical errors.

Fracture of endodontically treated teeth was reported as uncommon by 70.0% and common by 30.0% of respondents, with no respondents selecting ‘never’. These percentages correspond to the qualitative categories used in the questionnaire. Fracture of endodontically treated teeth was reported as uncommon by approximately 70% of respondents, while 25–30% considered it common and 3–5% described it as frequent.

## 4. Discussion

This large-scale survey confirms several global trends, such as the widespread use of NaOCl and rubber dam isolation, while also revealing region-specific patterns, including the notably high adoption of CBCT and the frequent use of sonic activation systems [1,12]. These findings build upon previous Latin American studies by quantifying the extent of technology uptake across multiple countries and by illustrating the uneven integration of newer endodontic technologies compared with Spain.

With 1358 valid responses, it represents one of the most extensive surveys conducted in these regions, allowing for meaningful comparisons with international data [1,3,10,12,15,16]. The demographic profile revealed a relatively young cohort, with nearly two-thirds having less than 10 years of experience, and most respondents reporting some form of postgraduate training. This supports the validity of the sample as a professional population actively engaged in endodontic care.

Magnification and imaging technologies showed a heterogeneous adoption pattern, showing a clear stratification according to the level of postgraduate education. More than half of the respondents reported using loupes, while nearly one-fifth reported routine use of the dental operating microscope. Although lower than the almost universal adoption reported by endodontists in Australia and New Zealand [10], these figures suggest a progressive integration of magnification into routine practice in Spain and Latin America. This trend contrasts with findings from undergraduate education, where Latin American dental schools, similar to those in regions outside North America and Western Europe, still rely predominantly on traditional teaching methods with limited incorporation of magnification [17,18]. Compared with data from 2010, these results suggest that, while exposure to magnification during formal training was very limited, its clinical adoption among practicing dentists has increased substantially [19].

The use of loupes was widespread across all training levels, indicating that basic magnification has become a standard component of modern endodontic care. However, the integration of the dental operating microscope remained largely confined to clinicians with structured postgraduate education, particularly those holding a ≥2-year master’s degree, among whom approximately one-third reported its routine use. In contrast, less than 10% of clinicians with only undergraduate training or short-course participation reported using a microscope, suggesting that advanced optical aids are still perceived as specialized tools rather than universal standards in clinical practice.

CBCT demonstrated the highest overall level of technological integration among all imaging tools evaluated. Its use was strikingly high, with over 90% of respondents reporting its application in selected cases. This percentage surpasses adoption rates documented in several European and North American surveys [10], indicating that CBCT has become an essential diagnostic tool in the region. Nearly all clinicians with master-level or current postgraduate education reported its use, compared with substantially lower adoption among general practitioners and those attending short courses. Moreover, postgraduate-trained respondents were more likely to employ CBCT for a broader range of diagnostic indications—including complex anatomy, retreatment, and surgical planning—whereas clinicians without structured training tended to limit its use to specific cases or lacked access entirely. These findings confirm that postgraduate education remains the main driver of CBCT adoption, surpassing years of experience or country of practice, and underscore its growing role as an essential diagnostic adjunct in contemporary endodontic decision-making [12,20,21].

NaOCl was overwhelmingly the irrigant of choice (98.3%), consistent with findings from other parts of the world [1,22]. However, the concentration varied markedly with education. Clinicians with master-level or structured postgraduate training more frequently reported using higher NaOCl concentrations (>2.5–5.25%) and combining them with adjunctive activation techniques, reflecting a more evidence-based approach to canal disinfection [23]. In contrast, lower concentrations were more common among respondents without postgraduate education. Chlorhexidine was used only sporadically and primarily by clinicians without advanced training, in line with its restricted role as an adjunct irrigant in specific clinical situations [24]. Such variations likely reflect differences in postgraduate training, clinical exposure, and access to modern irrigation technologies, which influence clinicians’ familiarity with concentration protocols and activation systems.

The use of adjunctive irrigation methods was also widespread, particularly sonic (83.2%) and ultrasonic activation (52.9%). These values are higher than those reported in Oceania and Europe, suggesting a rapid incorporation of activation systems [10]. Negative-pressure irrigation, however, remained rarely used, similar to other international surveys [10,22].

Activation systems were widely implemented, with sonic and ultrasonic methods reported at rates exceeding those observed in comparable European and Australian data [10]. Their use, however, was again education-dependent: postgraduate and master-level clinicians showed the highest adoption, often integrating both systems for improved irrigant penetration and smear-layer removal, whereas general practitioners relied primarily on manual irrigation. Similarly, the use of chelating agents such as EDTA was almost universal among trained endodontists but notably lower among non-specialists, where a subset still reported no smear-layer removal protocol. These patterns highlight the strong influence of postgraduate education on the standardization of irrigation strategies and on the incorporation of advanced technologies that enhance canal debridement and chemical cleaning efficacy.

The present study confirms the results from a recent survey conducted in Spain, which demonstrated that endodontists are more likely to use higher concentrations of NaOCl, routinely employ EDTA, and utilize adjunctive activation methods such as ultrasonic or sonic agitation to enhance irrigant efficacy [22]. The authors found no statistically significant differences in the choice of NaOCl, but they noted significant differences in the protocols used by general dentists and by endodontists in relation to its concentration, the use and type of irrigant used to remove the smear layer, the use of adjuncts to irrigation and the enlargement of the apical preparation when shaping a necrotic tooth, and the maintenance of apical patency throughout the debridement and shaping procedure. These differences highlight the impact of training and experience on the consistency and quality of irrigation practices.

In the present survey, hybrid manual–rotary techniques were predominant, whereas rotary-only instrumentation was mainly reported by clinicians with postgraduate training. This is consistent with international surveys showing that the adoption of NiTi rotary and reciprocating systems is strongly education-dependent and more frequent among trained endodontists than among general practitioners [10,11]. These parallels confirm that NiTi systems, although widely available, become fully integrated into routine practice primarily when supported by structured endodontic training.

Obturation preferences varied across regions and levels of training. Cold lateral condensation remained the most common technique, especially among clinicians with undergraduate or short-course training in countries such as Venezuela and Colombia. In contrast, warm vertical compaction was more frequently reported by master-level clinicians, reflecting greater exposure to advanced thermoplasticized methods through structured postgraduate education. Single-cone obturation accounted for approximately 15% of responses, while carrier-based systems were rare.

Sealer choice showed a similar gradient of modernization. Epoxy resin–based sealers (e.g., AH Plus) remained dominant overall, whereas bioceramic sealers were reported by 18.9% of participants, most commonly among those with postgraduate or master-level training, indicating their gradual but uneven clinical integration. Conversely, older materials such as zinc oxide–eugenol and calcium hydroxide–based sealers persisted mainly among practitioners without formal endodontic specialization.

The use of bioceramic sealers was still limited but clearly increased among clinicians with postgraduate training, indicating a gradual diffusion in the region. Similar trends were reported by Guivarc’h et al. [25], who observed broad international variability and frequent deviations from manufacturers’ recommendations. Consistent with the present data, general practitioners tended to favor the single-cone technique when using bioceramic sealers, while specialists and master-level clinicians more often employed thermoplasticized obturation methods. These findings suggest that the correct clinical use of bioceramic sealers remains closely linked to education and procedural familiarity rather than to material availability alone.

The higher use of bioceramic sealers in Venezuela may be related to factors outside the scope of this survey, such as recent emphasis on these materials in local training programs or differences in market availability. Although we cannot confirm this with the present data, these elements may help explain the trend observed in the regression analysis.

Secondary clinical practices revealed further insights into everyday endodontics. Rubber dam isolation was widely reported across all groups, marking clear progress compared with earlier surveys [26,27], yet its use remained inconsistent among general practitioners. While nearly all clinicians with structured postgraduate or master-level training reported routine use, adoption was notably lower among those without formal endodontic education, indicating that training continues to be the key determinant of adherence to standard isolation protocol. Previous data shows that in Spanish dental schools, rubber dam use is part of the curriculum and clinical training, but general dentists adoption drops significantly, mirroring trends seen across Latin America [13,17].

The adoption of single-visit root canal treatment in this survey closely mirrored respondents’ educational background. Master-trained clinicians were the most likely to perform treatment in a single appointment (75.4%), followed by those currently enrolled in postgraduate programs (64.1%) and those with modular postgraduate training (62.6%). In contrast, single-visit treatment was markedly less common among respondents whose training consisted solely of short courses (27.1%) or undergraduate education (43.9%). This gradient reinforces the central role of structured postgraduate education in shaping treatment philosophy, reinforcing confidence in contemporary disinfection protocols, and reducing reliance on multi-visit strategies. These findings align with international evidence indicating that clinicians with advanced training are more likely to integrate evidence-based, efficiency-oriented approaches into their routine practice [1,28,29].

Interestingly, the use of calcium hydroxide as an inter-appointment medicament did not vary as sharply across training categories as visit number did. Routine use ranged from 37% to 46% across all groups, while selective use in necrotic or retreatment cases remained the predominant pattern regardless of training level (45–53%). This suggests that the rationale for calcium hydroxide use is now largely standardized, driven more by case type than by practitioner experience or academic background [30,31]. Unlike single-visit treatment, which clearly reflects differences in education and confidence with modern irrigation adjuncts, calcium hydroxide use appears to be guided by clinical diagnosis rather than by training-related philosophy [32].

Maintenance of apical patency was consistently practiced by most respondents. Follow-up protocols varied, with around half scheduling routine evaluations within the first year, while others reported reviewing only if symptoms developed. Postoperative pain was generally described as uncommon, and fractures of endodontically treated teeth requiring extraction were considered infrequent. These findings highlight a generally conservative and symptom-driven approach to post-treatment monitoring [33], reflecting both regional practice patterns and the perceived predictability of contemporary endodontic procedures. However, the variability in follow-up routines suggests an opportunity to strengthen consensus on evidence-based recall intervals, which could improve long-term assessment of treatment outcomes and early identification of complications [34].

Regional comparisons highlighted differences in the pace of adoption of new technologies. Spain demonstrated higher integration of advanced methods such as warm vertical compaction, and microscope use. Mexico showed the highest prevalence of ultrasonic activation, while Venezuela and Colombia remained more conservative, with cold lateral condensation predominating. The demographic and educational composition of the respondents can partly explain the disparities observed between Spain and Latin American countries.

In Spain, a notably higher proportion of clinicians held structured postgraduate or master-level training (45.7% completed a ≥2-year master’s program and 27.9% a modular postgraduate), whereas in Venezuela and Colombia, only 12.3–24.1% reported master-level education, and a substantially larger fraction of respondents had undergraduate or short-course training (up to 49.4% in Venezuela and 21.3% in Colombia). This imbalance in the educational composition of the samples directly influences the apparent differences in technology adoption. Spanish respondents, who were predominantly specialists, reported higher use of microscopes (27.9% vs. 8.0–11.1% in Venezuela and Colombia, respectively), CBCT (96.8% vs. 82.7–92.6%), and warm vertical compaction (39.7% vs. 4.6–6.2%). These differences therefore reflect the profile of respondents in each country rather than intrinsic regional disparities in clinical philosophy or technological access.

When training level is evaluated independently of geography, clinicians with structured postgraduate education—whether practicing in Spain or Latin America—demonstrated remarkably similar clinical behaviors, including comparable use of magnification or irrigant activation. Conversely, respondents without formal postgraduate education across all countries showed more conservative approaches, such as reliance on cold lateral condensation (57.4% in Venezuela and 42.6% in Colombia) and reduced integration of advanced technologies. These patterns indicate that the observed country-level differences are primarily driven by the overrepresentation of general practitioners and early-stage clinicians in some regions, rather than by true geographic variability in endodontic practice.

Economic factors may also influence the adoption of CBCT and other advanced technologies. Across Spain and Latin America, both the cost of root canal treatment and the limited insurance coverage for CBCT examinations can restrict their routine use, leading clinicians to reserve these tools for selected cases. Although not directly evaluated in this survey, financial barriers are likely to contribute to the regional differences observed in technology uptake.

Comparisons with Asian data further contextualize the present findings. A recent survey reported that access to CBCT in the Asia–Pacific region remains substantially lower (65.4%) than in the Americas, suggesting persistent disparities in imaging availability [1]. Similarly, national data from India indicate considerable variability in the adoption of NiTi rotary systems, magnification, and working length determination, particularly among general practitioners [35]. Educational trends also mirror these differences: according to a recent study [17], more than half of Asian dental schools do not incorporate magnification into preclinical or clinical teaching, and many still rely on combined apex locator–radiographic methods for working length determination.

The strengths of this study include its large sample size, wide geographic coverage, and use of social media channels to capture responses from a younger and more digitally engaged population. Nevertheless, some limitations must be acknowledged.

Convenience sampling through social media recruitment likely introduced selection bias, favoring younger and digitally engaged professionals, and may limit generalizability. Indeed, evidence from recent surveys [36,37] show that social media platforms used for professional communication are predominantly adopted by younger dental practitioners, who exhibit higher levels of online engagement than older colleagues. This well-documented pattern in digital health behavior helps explain the younger profile of these respondents, given that the survey was distributed through these platforms.

In addition, the aggregation of diverse countries into the ‘Other countries’ category oversimplifies contextual differences. The ‘Other countries’ category included respondents from Latin American nations not represented in the main subgroups, such as Brazil, Costa Rica, Panama, Guatemala, Honduras, El Salvador, Nicaragua, Paraguay, Uruguay, the Dominican Republic, Puerto Rico, and Cuba. Because these countries differ substantially in their socio-economic and educational contexts, the heterogeneity of this cluster should be considered when interpreting regional comparisons.

The inability to calculate a response rate is a further limitation that should be considered when interpreting the results.

Overall, this survey provides a clear picture of the diffusion of innovation in endodontics across Spain and Latin America. While sodium hypochlorite irrigation and rubber dam isolation are well-established, advanced technologies such as microscopes and bioceramic sealers are still in a phase of gradual adoption, with Spain showing faster integration than many Latin American countries. These findings underscore the importance of strengthening postgraduate training and continuing education initiatives to promote evidence-based practice and reduce disparities in the uptake of endodontic innovations across the region.

## 5. Conclusions

Endodontic practice across Spain and Latin America shows strong adherence to fundamental standards, such as NaOCl irrigation and rubber dam isolation, yet the integration of advanced technologies remains uneven. CBCT, magnification, and bioceramic sealers were mainly adopted by clinicians with postgraduate or master-level training, emphasizing the decisive role of education in shaping evidence-based practice. Strengthening postgraduate programs is essential to harmonize clinical standards and foster consistent technological advancement across the region.

## Figures and Tables

**Table 1:** Demographic characteristics of the population under study. Distribution of the year of experience and endodontics training level.

Variable	Spain	Mexico	Venezuela	Colombia	Southern Cone & Andes	Other Countries	Total
1–5 years	106 (48.4%)	174 (49.3%)	43 (26.5%)	36 (33.3%)	88 (33.3%)	95 (37.7%)	542 (39.9%)
6–10 years	58 (26.5%)	75 (21.2%)	36 (22.2%)	21 (19.4%)	69 (26.1%)	52 (20.6%)	311 (22.9%)
11–20 years	34 (15.5%)	54 (15.3%)	40 (24.7%)	31 (28.7%)	64 (24.2%)	59 (23.4%)	282 (20.8%)
>20 years	21 (9.6%)	50 (14.2%)	43 (26.5%)	20 (18.5%)	43 (16.3%)	46 (18.3%)	223 (16.4%)
Undergraduate/ graduate only	16 (7.3%)	32 (9.1%)	25 (15.4%)	23 (21.3%)	23 (8.7%)	36 (14.3%)	155 (11.4%)
Courses/ congresses	15 (6.8%)	42 (11.9%)	80 (49.4%)	9 (8.3%)	50 (18.9%)	44 (17.5%)	240 (17.7%)
Modular postgraduate	61 (27.9%)	124 (35.1%)	28 (17.3%)	45 (41.7%)	82 (31.1%)	53 (21.0%)	393 (28.9%)
Master ≥ 2 years	100 (45.7%)	122 (34.6%)	20 (12.3%)	26 (24.1%)	83 (31.4%)	88 (34.9%)	439 (32.3%)
Currently in postgraduate program	27 (12.3%)	33 (9.3%)	9 (5.6%)	5 (4.6%)	26 (9.8%)	31 (12.3%)	131 (9.6%)

**Table 2:** Clinical practice and technology adoption by country group. Values are n (%). Percentages rounded to one decimal place.

Variable	Spain	Mexico	Venezuela	Colombia	Southern Cone & Andes	Other Countries	Total
Magnification							
Microscope	61 (27.9%)	71 (20.1%)	13 (8.0%)	12 (11.1%)	46 (17.1%)	49 (19.8%)	252 (18.6%)
Loupes	100 (45.7%)	200 (56.7%)	105 (64.8%)	63 (58.3%)	144 (53.5%)	140 (56.7%)	752 (55.4%)
None	58 (26.5%)	82 (23.2%)	44 (27.2%)	33 (30.6%)	79 (29.4%)	58 (23.5%)	354 (26.1%)
Irrigant of choice							
NaOCl	211 (96.3%)	348 (98.6%)	158 (97.5%)	107 (99.1%)	267 (99.3%)	244 (98.8%)	1335 (98.3%)
Chlorhexidine	7 (3.2%)	0 (0.0%)	3 (1.9%)	0 (0.0%)	2 (0.7%)	1 (0.4%)	13 (1.0%)
EDTA	1 (0.5%)	4 (1.1%)	1 (0.6%)	0 (0.0%)	0 (0.0%)	0 (0.0%)	6 (0.4%)
Other	0 (0.0%)	1 (0.3%)	0 (0.0%)	1 (0.9%)	0 (0.0%)	2 (0.8%)	4 (0.3%)
Irrigation adjunct							
Ultrasonic	69 (31.5%)	243 (68.8%)	86 (53.1%)	28 (25.9%)	157 (58.4%)	136 (55.1%)	719 (52.9%)
Sonic	181 (82.6%)	320 (90.7%)	111 (68.5%)	88 (81.5%)	221 (82.2%)	209 (84.6%)	1130 (83.2%)
Negative pressure	5 (2.3%)	12 (3.4%)	13 (8.0%)	5 (4.6%)	11 (4.1%)	8 (3.2%)	54 (4.0%)
None	16 (7.3%)	4 (1.1%)	8 (4.9%)	5 (4.6%)	14 (5.2%)	6 (2.4%)	53 (3.9%)
Obturation technique							
Warm vertical	87 (39.7%)	47 (13.3%)	10 (6.2%)	5 (4.6%)	30 (11.2%)	35 (14.2%)	214 (15.8%)
Cold lateral	20 (9.1%)	117 (33.1%)	93 (57.4%)	46 (42.6%)	106 (39.4%)	80 (32.4%)	462 (34.0%)
Single cone	28 (12.8%)	61 (17.3%)	21 (13.0%)	20 (18.5%)	37 (13.8%)	44 (17.8%)	211 (15.5%)
Carrier-based	16 (7.3%)	0 (0.0%)	0 (0.0%)	0 (0.0%)	1 (0.4%)	3 (1.2%)	20 (1.5%)
Sealer type							
Bioceramic	22 (10.0%)	74 (21.0%)	50 (30.9%)	16 (14.8%)	44 (16.4%)	51 (20.6%)	257 (18.9%)
Epoxy-resin	122 (55.7%)	116 (32.9%)	41 (25.3%)	54 (50.0%)	108 (40.1%)	88 (35.6%)	529 (39.0%)
Other	75 (34.2%)	166 (47.0%)	71 (43.8%)	39 (36.1%)	118 (43.9%)	109 (44.1%)	578 (42.6%)
CBCT usage							
Yes	212 (96.8%)	323 (91.5%)	134 (82.7%)	100 (92.6%)	252 (93.7%)	232 (93.9%)	1253 (92.3%)
No	7 (3.2%)	30 (8.5%)	28 (17.3%)	8 (7.4%)	17 (6.3%)	15 (6.1%)	105 (7.7%)

**Table 3:** Clinical Variables by type of training levels. Values are n (%). Percentages rounded to one decimal place.

Variable	Category	Currently Postgraduate/ Master (n = 131)	Courses, Congresses (n = 240)	Bachelor’s Degree (n = 155)	Master (≥2 Years) (n = 439)	Modular Postgraduate (n = 393)
Rubber dam	Always	128 (97.7%)	186 (77.5%)	121 (78.1%)	433 (98.6%)	366 (93.1%)
	Occasionally	3 (2.3%)	53 (22.1%)	25 (16.1%)	5 (1.1%)	27 (6.9%)
	Never	0 (0.0%)	1 (0.4%)	9 (5.8%)	1 (0.2%)	0 (0.0%)
Endodontic sessions	One session (if possible)	84 (64.1%)	65 (27.1%)	68 (43.9%)	331 (75.4%)	246 (62.6%)
	Two sessions	47 (35.9%)	175 (72.9%)	87 (56.1%)	108 (24.6%)	147 (37.4%)
Obturation system	Lateral compaction	33 (25.2%)	136 (56.7%)	80 (51.6%)	84 (19.1%)	129 (32.8%)
	Vertical compaction	27 (20.6%)	10 (4.2%)	11 (7.1%)	103 (23.5%)	63 (16.0%)
	Single cone	20 (15.3%)	47 (19.6%)	25 (16.1%)	52 (11.9%)	67 (17.1%)
	All depending on the case	49 (37.4%)	41 (17.1%)	35 (22.6%)	196 (44.7%)	130 (33.1%)
	Carrier-based	2 (1.5%)	6 (2.5%)	4 (2.6%)	4 (0.9%)	4 (1.0%)
Sealer Type	Resin-based	50 (38.2%)	84 (35.0%)	69 (44.5%)	165 (37.6%)	162 (41.2%)
	Bioceramics	25 (19.1%)	63 (26.3%)	28 (18.1%)	83 (18.9%)	84 (21.4%)
	Zinc oxide–eugenol–based	5 (3.8%)	9 (3.8%)	6 (3.9%)	6 (1.4%)	5 (1.3%)
	Mixed/other/depends	51 (38.9%)	84 (35.0%)	52 (33.5%)	185 (42.1%)	142 (36.1%)
Working length measurement	Both (apex locator + radiograph)	90 (68.7%)	128 (53.3%)	74 (47.7%)	230 (52.4%)	226 (57.5%)
	Apex locator only	41 (31.3%)	53 (22.1%)	40 (25.8%)	201 (45.8%)	150 (38.2%)
	Radiographically only	0 (0.0%)	57 (23.8%)	41 (26.5%)	8 (1.8%)	17 (4.3%)
Instrumentation type	Combined (manual + rotary)	118 (90.1%)	158 (65.8%)	88 (56.8%)	399 (90.9%)	339 (86.3%)
	Manual only	5 (3.8%)	67 (27.9%)	58 (37.4%)	1 (0.2%)	10 (2.5%)
	Rotary only	8 (6.1%)	15 (6.3%)	9 (5.8%)	39 (8.9%)	44 (11.2%)
Chlorhexidine concentration	0.05% (0.0005)	2 (1.5%)	5 (2.1%)	3 (1.9%)	—	6 (1.5%)
	0.12% (0.0012)	10 (7.6%)	36 (15.0%)	16 (10.3%)	37 (8.4%)	27 (6.9%)
	2% (0.02)	41 (31.3%)	80 (33.3%)	41 (26.5%)	138 (31.4%)	133 (33.8%)
	Do not use chlorhexidine	78 (59.5%)	119 (49.6%)	95 (61.3%)	264 (60.1%)	227 (57.8%)
NaOCl concentration	0.5–2.5%	16 (12.2%)	65 (27.1%)	40 (25.8%)	50 (11.4%)	62 (15.8%)
	<0.5%	5 (3.8%)	17 (7.1%)	14 (9.0%)	4 (0.9%)	11 (2.8%)
	>2.5–5.25%	107 (81.7%)	153 (63.8%)	91 (58.7%)	382 (87.0%)	316 (80.4%)
	Unknown concentration	3 (2.3%)	5 (2.1%)	10 (6.5%)	3 (0.7%)	4 (1.0%)
Primary irrigant	Chlorhexidine	1 (0.8%)	6 (2.5%)	1 (0.6%)	4 (0.9%)	1 (0.3%)
	EDTA	1 (0.8%)	1 (0.4%)	1 (0.6%)	0 (0.0%)	3 (0.8%)
	NaOCl (sodium hypochlorite)	127 (97.0%)	231 (96.3%)	153 (98.7%)	435 (99.1%)	389 (99.0%)
	Other	2 (1.5%)	2 (0.8%)	0 (0.0%)	0 (0.0%)	0 (0.0%)
Activation	Ultrasonic activation (PUI, passive ultrasonic)	84 (64.1%)	118 (49.2%)	53 (34.2%)	246 (56.0%)	216 (55.0%)
	Sonic activation	38 (29.0%)	44 (18.3%)	46 (29.7%)	153 (34.9%)	129 (32.8%)
	Negative apical pressure	3 (2.3%)	16 (6.7%)	10 (6.5%)	10 (2.3%)	14 (3.6%)
	None/ manual/other	6 (4.6%)	62 (25.8%)	46 (29.7%)	30 (6.8%)	34 (8.7%)
Magnification system	Loupes	84 (64.1%)	143 (59.6%)	65 (41.9%)	235 (53.5%)	225 (57.3%)
	Microscope	22 (16.8%)	8 (3.3%)	14 (9.0%)	149 (33.9%)	59 (15.0%)
	None	25 (19.1%)	89 (37.1%)	76 (49.0%)	55 (12.5%)	109 (27.7%)
CBCT Indications	Multiple indications (all/most of the above; generic/other)	102 (77.9%)	115 (47.9%)	81 (52.3%)	346 (78.8%)	272 (69.2%)
	Complex anatomy (incl. complex cases)	12 (9.2%)	29 (12.1%)	15 (9.7%)	36 (8.2%)	52 (13.2%)
	Retreatment	5 (3.8%)	23 (9.6%)	18 (11.6%)	13 (3.0%)	22 (5.6%)
	Apical surgery	8 (6.1%)	10 (4.2%)	17 (11.0%)	29 (6.6%)	27 (6.9%)
	None/do not use/no access/don’t know	4 (3.1%)	63 (26.3%)	24 (15.5%)	15 (3.4%)	20 (5.1%)
clinical time in endodontics	100% (exclusive endodontist)	29 (22.1%)	11 (4.6%)	29 (18.7%)	244 (55.6%)	145 (36.9%)
	<50%	38 (29.0%)	131 (54.6%)	73 (47.1%)	32 (7.3%)	93 (23.7%)
	>50%	64 (48.9%)	98 (40.8%)	53 (34.2%)	163 (37.1%)	155 (39.4%)
Years of experience in endodontics	1–5 years	115 (87.8%)	80 (33.3%)	80 (51.6%)	126 (28.7%)	141 (35.9%)
	6–10 years	13 (9.9%)	60 (25.0%)	23 (14.8%)	106 (24.1%)	109 (27.7%)
	11–20 years	2 (1.5%)	48 (20.0%)	27 (17.4%)	114 (26.0%)	91 (23.2%)
	>20 years	1 (0.8%)	52 (21.7%)	25 (16.1%)	93 (21.2%)	52 (13.2%)

**Table 4:** Logistic regression analyses regarding bioceramic sealer use (adjusted odds ratios, 95% CI).

Predictor	OR (95% CI)	z	*p*-Value
Country: Mexico	1.52 (0.84–2.74)	1.38	0.169
Country: Other countries	1.49 (0.81–2.76)	1.28	0.201
Country: Southern Cone & Andes	1.12 (0.60–2.09)	0.37	0.714
Country: Spain	0.64 (0.32–1.28)	−1.27	0.204
Country: Venezuela	2.56 (1.35–4.84)	2.89	0.004
Training: Postgraduate	0.98 (0.72–1.34)	−0.12	0.908
Experience (per category increase)	0.98 (0.86–1.11)	−0.32	0.749

Note: Bioceramic sealer use (1 = yes).

**Table 5:** Logistic regression analyses regarding CBCT adoption (adjusted odds ratios, 95% CI).

Predictor	OR (95% CI)	z	*p*-Value
Country: Mexico	0.71 (0.30–1.64)	−0.81	0.418
Country: Other countries	1.31 (0.53–3.27)	0.58	0.559
Country: Southern Cone & Andes	1.15 (0.47–2.81)	0.30	0.766
Country: Spain	1.75 (0.60–5.10)	1.02	0.308
Country: Venezuela	0.65 (0.27–1.53)	−1.00	0.319
Training: Postgraduate	5.72 (3.63–9.02)	7.50	0.000
Experience (per category increase)	1.01 (0.84–1.22)	0.11	0.909

Note: CBCT adoption (1 = yes).

**Table 6:** Secondary variables: clinical practice patterns by country group.

Variable	Spain	Mexico	Venezuela	Colombia	Southern Cone & Andes	Other Countries	Total
Rubber dam							
Always	217 (99.1%)	353 (100.0%)	160 (98.8%)	106 (98.1%)	266 (98.9%)	245 (99.2%)	1347 (99.2%)
Not always	0 (0.0%)	0 (0.0%)	0 (0.0%)	0 (0.0%)	0 (0.0%)	0 (0.0%)	0 (0.0%)
Treatment sessions							
Single visit	185 (84.5%)	178 (50.4%)	38 (23.5%)	85 (78.7%)	165 (61.3%)	143 (57.9%)	794 (58.5%)
Multiple visits	34 (15.5%)	175 (49.6%)	124 (76.5%)	23 (21.3%)	104 (38.7%)	104 (42.1%)	564 (41.5%)
Calcium hydroxide							
Yes	170 (77.6%)	253 (71.7%)	99 (61.1%)	82 (75.9%)	183 (68.0%)	167 (67.6%)	954 (70.3%)
No	3 (1.4%)	0 (0.0%)	2 (1.2%)	0 (0.0%)	4 (1.5%)	2 (0.8%)	11 (0.8%)
Apical patency							
Yes	69 (31.5%)	156 (44.2%)	108 (66.7%)	64 (59.3%)	160 (59.5%)	125 (50.6%)	682 (50.2%)
No	1 (0.5%)	9 (2.5%)	5 (3.1%)	1 (0.9%)	7 (2.6%)	9 (3.6%)	32 (2.4%)
Follow-up							
≤6 months	116 (53.0%)	134 (38.0%)	63 (38.9%)	45 (41.7%)	91 (33.8%)	106 (42.9%)	555 (40.9%)
1 year	11 (5.0%)	4 (1.1%)	3 (1.9%)	0 (0.0%)	4 (1.5%)	5 (2.0%)	27 (2.0%)
Only if symptoms	0 (0.0%)	0 (0.0%)	0 (0.0%)	0 (0.0%)	0 (0.0%)	1 (0.4%)	1 (0.1%)
Postoperative pain							
Common	0 (0.0%)	0 (0.0%)	0 (0.0%)	0 (0.0%)	0 (0.0%)	0 (0.0%)	0 (0.0%)
Uncommon	166 (75.8%)	321 (90.9%)	146 (90.1%)	92 (85.2%)	240 (89.2%)	208 (84.2%)	1173 (86.4%)
Never	0 (0.0%)	0 (0.0%)	0 (0.0%)	0 (0.0%)	0 (0.0%)	0 (0.0%)	0 (0.0%)
Fracture after RCT							
Common	0 (0.0%)	0 (0.0%)	0 (0.0%)	0 (0.0%)	0 (0.0%)	0 (0.0%)	0 (0.0%)
Uncommon	125 (57.1%)	236 (66.9%)	119 (73.5%)	84 (77.8%)	214 (79.6%)	172 (69.6%)	950 (70.0%)
Never	0 (0.0%)	0 (0.0%)	0 (0.0%)	0 (0.0%)	0 (0.0%)	0 (0.0%)	0 (0.0%)

Note: Some variables allowed multiple responses.

**Table 7:** Secondary variables: clinical practice patterns by training level.

Variable	Category	Currently Postgraduate/ Master (n = 131)	Courses, Congresses (n = 240)	Bachelor’s Degree (n = 155)	Master (≥2 Years) (n = 439)	Modular Postgraduate (n = 393)
Rubber dam	Always	128 (97.7%)	186 (77.5%)	121 (78.1%)	433 (98.6%)	366 (93.1%)
	Occasionally	3 (2.3%)	53 (22.1%)	25 (16.1%)	5 (1.1%)	27 (6.9%)
	Never	0 (0.0%)	1 (0.4%)	9 (5.8%)	1 (0.2%)	0 (0.0%)
Treatment sessions	Single visit	84 (64.1%)	65 (27.1%)	68 (43.9%)	331 (75.4%)	246 (62.6%)
	Multiple visits	47 (35.9%)	175 (72.9%)	87 (56.1%)	108 (24.6%)	147 (37.4%)
Patency maintenance	As often as possible	47 (82.5%)	132 (91.0%)	73 (82.0%)	196 (89.5%)	190 (89.6%)
	Always	2 (3.5%)	1 (0.7%)	0 (0.0%)	2 (0.9%)	4 (1.9%)
	Never/not important	8 (14.0%)	10 (6.9%)	16 (18.0%)	12 (5.5%)	16 (7.5%)
	Other/depends	0 (0.0%)	2 (1.4%)	0 (0.0%)	9 (4.1%)	2 (0.9%)
Pre-operative x-Ray	Always	37 (64.9%)	66 (45.5%)	44 (49.4%)	134 (61.2%)	113 (53.3%)
	Sometimes (if in doubt)	20 (35.1%)	70 (48.3%)	38 (42.7%)	82 (37.4%)	96 (45.3%)
	Never	0 (0.0%)	9 (6.2%)	7 (7.9%)	1 (0.5%)	3 (1.4%)
Calcium Hydroxide	Always	52 (39.7%)	111 (46.3%)	71 (45.8%)	164 (37.4%)	171 (43.5%)
	Only if necrotic/retreatment/suppuration	70 (53.4%)	109 (45.4%)	75 (48.4%)	221 (50.3%)	190 (48.3%)
	Case-dependent/other conditional	8 (6.1%)	8 (3.3%)	7 (4.5%)	42 (9.6%)	23 (5.9%)
	Never	1 (0.8%)	12 (5.0%)	2 (1.3%)	12 (2.7%)	9 (2.3%)
Working length measurements	Both (apex locator + radiograph)	90 (68.7%)	128 (53.3%)	74 (47.7%)	230 (52.4%)	226 (57.5%)
	Apex locator only	41 (31.3%)	53 (22.1%)	40 (25.8%)	201 (45.8%)	150 (38.2%)
	Radiographically only	0 (0.0%)	57 (23.8%)	41 (26.5%)	8 (1.8%)	17 (4.3%)
Postoperative pain	Uncommon	107 (81.7%)	213 (88.8%)	131 (84.5%)	374 (85.2%)	348 (88.6%)
	Common	21 (16.0%)	20 (8.3%)	24 (15.5%)	61 (13.9%)	41 (10.4%)
	Frequent	3 (2.3%)	7 (2.9%)	0 (0.0%)	4 (0.9%)	4 (1.0%)
Fracture after RCT	Uncommon	93 (71.0%)	174 (72.5%)	108 (69.7%)	280 (63.8%)	295 (75.1%)
	Common	34 (26.0%)	60 (25.0%)	41 (26.5%)	143 (32.6%)	78 (19.9%)
	Frequent	4 (3.1%)	6 (2.5%)	6 (3.9%)	16 (3.6%)	20 (5.1%)
Endodontic success	Both clinical & radiographic	115 (87.8%)	192 (80.0%)	133 (85.8%)	394 (89.8%)	362 (92.1%)
	Radiographic only	1 (0.8%)	5 (2.1%)	3 (1.9%)	6 (1.4%)	3 (0.8%)
	Clinical only (no signs/symptoms)	15 (11.5%)	41 (17.1%)	18 (11.6%)	38 (8.7%)	28 (7.1%)
	None of the above	0 (0.0%)	2 (0.8%)	1 (0.6%)	1 (0.2%)	0 (0.0%)

## Data Availability

The original contributions presented in this study are included in the article. Further inquiries can be directed to the corresponding author.
